# Using structure- and Ligand-based pharmacophores as filters to discriminate Human Aryl Sulfotransferase 1A1 (SUL1A1) binders into substrates and inhibitors

**DOI:** 10.1186/1758-2946-6-S1-P32

**Published:** 2014-03-11

**Authors:** Salwa M Soliman, Gerhard Wolber

**Affiliations:** 1 Freie Universitaet Berlin, Institute of Pharmacy, Department pharmaceutical, chemistry, Koenigin-Luisestrasse 2+4, 14195 Berlin, Germany

## 

Predicting metabolictransformation is one of major challenges in drug discovery [1]. Sulfotransferase 1A1 (SULT1A1), one of phase II metabolismenzymes, is the major SULT in adult liver catalysis [2]. It metabolizes many endogenous compounds and is relevant in carcinogenesis due to its ability to modify diverse promutagen and procarcinogen xenobiotics [3].

In order to make a discriminative model that classifies group of SULT1A1 binders into substrates and inhibitors, a combination of structure, ligand-based, and docking based pharmacophores have been generated and validated by Ligandscout [4].

On one hand, structure-based pharmacophores have been derived from PDB files of good binders (substrates and inhibitors). On the other hand, ligand-based interaction maps have been conducted from some drug classes that show different substrate/inhibitor activity towards SULT1A1.Finally, highly active substrates and inhibitors have been docked into the enzyme using GOLD [5], and subsequently molecular interaction fields have been developed for the most plausible poses.

As a retrospective validation, all pharmacophores simultaneously have been used to screen more than 100 SULT1A1 binders covering several activity classes and different chemical scaffolds. The model showed good discriminative power to differentiate between inhibitors, substrates and mixed substrates/inhibitors.
